# The Natural History of Prediabetes and Cardiovascular Disease in the Pediatric Population

**DOI:** 10.3390/biomedicines14010198

**Published:** 2026-01-16

**Authors:** Siham Accacha, Julia Barillas-Cerritos, Liana Gabriel, Ankita Srivastava, Shelly Gulkarov, Jennifer A. Apsan, Joshua De Leon, Allison B. Reiss

**Affiliations:** 1 Department of Pediatrics, NYU Grossman Long Island School of Medicine, Mineola, NY 11501, USA; saccacha@northwell.edu (S.A.); julia.barillascerritos@nyulangone.org (J.B.-C.); liana.gabriel@nyulangone.org (L.G.); 2 Department of Pediatrics, Donald and Barbara Zucker School of Medicine at Hofstra/Northwell, Hempstead, NY 11549, USA; japsan@northwell.edu; 3 Department of Foundations of Medicine, NYU Grossman Long Island School of Medicine, Mineola, NY 11501, USA; ankita.srivastava@nyulangone.org (A.S.); shelly.gulkarov@nyulangone.org (S.G.); 4 Department of Medicine, NYU Grossman Long Island School of Medicine, Mineola, NY 11501, USA; joshua.deleon@nyulangone.org

**Keywords:** prediabetes, type 2 diabetes mellitus, adolescents, children, obesity, cardiovascular risk factors, β-cell function, incretin, insulin resistance

## Abstract

The prevalence and incidence of prediabetes in children and youth continue to increase in parallel with the obesity epidemic. While prediabetes is defined by elevated HbA1c and/or impaired glucose tolerance (IGT) and/or impaired fasting glucose (IFG), the risk of clinical disease is a continuum. Individuals with prediabetes are at a higher risk of developing youth-onset type 2 diabetes, which is considered a more aggressive form of the disease. This condition is associated with increased cardiovascular and metabolic risks and leads to an earlier onset of complications compared to adults with type 2 diabetes. Additionally, significant damage to beta cells may occur even before dysglycemia develops. Recent data indicate that mortality rates are higher in youths with type 2 diabetes compared to those with type 1 diabetes. Childhood prediabetes and cardiovascular complications associated with it are a significant health concern. This review provides the latest insights into this complex issue. We will present an overview of pathophysiology, screening methods, and therapeutic options to prevent the progression from prediabetes to type 2 diabetes in children. In summary, it is crucial to identify prediabetes in children, as this underscores the importance of appropriate screening and timely intervention.

## 1. Introduction

Prediabetes is a condition characterized by abnormal carbohydrate metabolism and is viewed as a continuum that can progress to type 2 diabetes [1,2,3]. Prediabetes is linked to a higher rate of all-cause morbidity and mortality [3]. Prediabetes affected approximately 720 million individuals worldwide in 2021 and will affect an estimated 1 billion people by 2045 according to the International Diabetes Federation (IDF) [4].

Prediabetes is characterized by a glycated hemoglobin (HbA1c) level between 5.7% and 6.4%, a fasting serum glucose level ranging from 100 to 125 mg/dL, and/or impaired glucose tolerance (IGT) [5,6]. However, these various diagnostic criteria do not capture all individuals at high risk [7,8]. Additionally, the cutoff points established by different organizations are inconsistent and may vary for diagnosing prediabetes based on ethnicity [9].

While prediabetes is typically asymptomatic, individuals with this condition frequently exhibit obesity, dyslipidemia, and hypertension. Prediabetes is acknowledged as a high-risk metabolic state for developing both diabetes and cardiovascular disease (CVD) [10,11,12,13]. Numerous epidemiological studies indicate that prediabetes is a strong predictor of CVD, with the risk beginning to rise well before diabetes develops [14]. Recent studies on adults have shown, with moderate certainty, that prediabetes is positively linked to an increased risk of all-cause mortality and the occurrence of cardiovascular issues, including coronary heart disease (CHD) and stroke [10,15,16]. Major knowledge gaps exist regarding the natural history of prediabetes in youth, and there is a clear need to determine the effectiveness of interventions on cardiovascular outcomes.

The present review provides a broad summary of the interconnections among prediabetes, oxidative stress, insulin resistance, and CVD in the pediatric population. It focuses on how insulin resistance and oxidative stress in prediabetes may play a role in causing metabolic alterations that promote the development of CVD. In addition, a comprehensive overview of the most up-to-date therapeutic approaches in the pediatric population is highlighted.

## 2. Methodology

A comprehensive literature search was conducted with the aim of capturing current relevant evidence pertaining to prediabetes and CVD in adolescents and children and their interconnection. The search encompassed five electronic databases, including PubMed, Scopus, Embase, Web of Science, and Google Scholar. Peer-reviewed literature in English from the past 15 years was included in the initial screen with the most recent papers prioritized. Key terms used in various combinations included “prediabetes”, “obesity”, “diabetes mellitus”, “cardiovascular disease”, “childhood,” adolescent”, “treatment”, “risk”, “risk factor”, “GLP-1 receptor agonists”, “insulin resistance”, “weight loss”, and “clinical care pathways”. Keywords were then refined based on the relevance of the results, and additional terms were searched to survey related areas including “cardiometabolic risk factors”, “body mass index (BMI)”, “pathophysiology” and “incretin”. Articles were initially screened for relevance based on the content of their abstracts, followed by a full-text evaluation to select those that provided the most accurate and up-to-date information. The search was updated iteratively during preparation of the manuscript to incorporate newly published data. This review primarily summarizes evidence relating to children and adolescents under the age of 18 years but was extended to adults over the age of 18 years with clear demarcation when information was limited in the core age group.

## 3. Defining and Identifying Risk Factors for Prediabetes in the Pediatric Population

### 3.1. Defining Prediabetes in Children

The criteria used to diagnose prediabetes in children after the onset of puberty or above age 10 years is the same criteria used in adults [17,18,19,20,21]. No criteria exist for younger children and pertinent testing is based on clinical judgement. The American Diabetes Association and the American Academy of Pediatrics have defined prediabetes in children and adolescents (<18 years) [22].

Prediabetes is diagnosed if any of the following are present:

- Impaired fasting plasma glucose (IFG): 100–125 mg/dL.
- 2-h plasma glucose during an oral glucose tolerance test: 140–199 mg/dL.
- Hemoglobin A1c (HbA1c): 5.7% to 6.4%.

These criteria are applied after the onset of puberty or age 10, whichever comes first, in youth with overweight/obesity and additional risk factors for diabetes [18,19].

The societies note that the epidemiological studies supporting these thresholds were conducted in adults, and the sensitivity and specificity of these tests, especially HbA1c, may be lower in children. The American Academy of Pediatrics recommends using HbA1c for monitoring prediabetes but highlights that fasting glucose and oral glucose tolerance test criteria are also acceptable and may be more sensitive in some pediatric populations [17]. Both societies emphasize that the concordance between fasting plasma glucose, oral glucose tolerance test, and HbA1c is imperfect in children, and clinical judgment should guide test selection and interpretation.

### 3.2. Diagnosing Prediabetes in Children

Wallace et al. provide a comprehensive evaluation of screening and diagnostic strategies for prediabetes and diabetes in US children and adolescents using NHANES data from 1999–2016 [20]. The study found that one-quarter of U.S. youth are eligible for diabetes or prediabetes screening; however, few actually test positive, particularly for diabetes. The HbA1c test, which is a specific, non-fasting method for identifying high-risk youth, has limited sensitivity. Most youth with diabetes have already been diagnosed, which means the current screening criteria may overlook some at-risk individuals. The study supports the idea of targeted screening and advocates for the use of HbA1c to identify youth who could benefit from preventive interventions.

Building on these diagnostic thresholds, recent epidemiological studies and expert reviews highlight several important considerations regarding the application and performance of these criteria in pediatric populations. Large cross-sectional analyses of US adolescents confirm that the prevalence of prediabetes—defined by impaired fasting glucose, impaired glucose tolerance, or elevated HbA1c—has increased in recent years, with approximately 17% of adolescents aged 12–19 years meeting at least one criterion [23]. Andes et al. report that 18% of adolescents have prediabetes and 11.1% have impaired fasting glucose [23]. They also found that male adolescents have significantly higher prediabetes prevalence (22.5%) compared to females (13.4%). Adolescents and young adults with prediabetes demonstrate an unfavorable cardiometabolic risk profile, including significantly higher non-HDL cholesterol, systolic blood pressure, central adiposity, and lower insulin sensitivity compared to those with normal glucose tolerance. This places them at increased risk for both type 2 diabetes and CVD. A global meta-analysis from Han et al. found that the pooled prevalence of prediabetes in children and adolescents was 8.8% with higher prevalence in males than females [24]. There was also a higher prevalence of prediabetes in children with a family history of diabetes, those with higher BMI and those who lived in an urban versus rural setting. Finally, there was a temporal uptrend with prevalence increasing from 0.93% to 10.66% over past decades.

Research demonstrates that HbA1c, while highly specific, has lower sensitivity for identifying youth with cardiometabolic risk compared to fasting plasma glucose or oral glucose tolerance testing. For example, in Wallace et al.’s study, a nationally representative sample, HbA1c ≥ 5.7% was highly specific (98.6%) but had low sensitivity (4.0%) for detecting high-risk youth, whereas fasting glucose ≥ 100 mg/dL was more sensitive (19.4%) but less specific (90.1%) [20]. This underscores the importance of considering multiple testing modalities and clinical context when evaluating prediabetes in children. Additionally, population-based studies emphasize that the risk factors for prediabetes in youth—such as obesity, family history, and certain racial/ethnic backgrounds—are similar to those in adults, but the natural history and progression to type 2 diabetes may differ, with some youth reverting to normoglycemia over time [23]. These findings support the need for individualized risk assessment and repeat testing to confirm abnormal results before making a diagnosis or initiating interventions.

### 3.3. Risk Factors Predisposing Individuals to Prediabetes in the Pediatric Population

The major risk factors for developing prediabetes in children and adolescents (under age 18 years) are obesity (especially central adiposity), family history of type 2 diabetes, minority race/ethnicity, exposure to maternal diabetes or gestational diabetes, and signs of insulin resistance [25]. These risk factors are well-established in the medical literature and are supported by multiple guidelines and epidemiological studies.

Obesity is the strongest modifiable risk factor, with higher body mass index (BMI) and central adiposity significantly increasing risk [17,18,26]. Family history of type 2 diabetes in first- or second-degree relatives, and maternal history of diabetes or gestational diabetes, are important non-modifiable risk factors [17,18,22,27]. Minority race/ethnicity—including American Indian/Alaska Native, Black, and Hispanic/Latino youth—are associated with a substantially higher prevalence of prediabetes, likely due to a combination of genetic, environmental, and structural factors [22,28,29].

Additional risk factors include signs of insulin resistance (such as acanthosis nigricans, hypertension, dyslipidemia, polycystic ovary syndrome, and abnormal birth weight), use of obesogenic psychotropic medications, and early or rapid pubertal development, which is associated with transient physiologic insulin resistance and socioeconomic factors [17,18,22,28,29]. Chronic stress, and sleep disorders may also contribute [30,31]. Table 1 summarizes the risk factors that are associated with pre-diabetes in children and adolescents.

#### 3.3.1. Lifestyle and Obesity

There is evidence that intensive lifestyle modification in obese children with prediabetes can improve markers of insulin resistance [32]. There is a large body of evidence pointing to obesity as a major risk factor for diabetes and prediabetes. The article by Ouyang, Hu, and Chen provides a comprehensive analysis of national trends and risk factors for diabetes and prediabetes among US adolescents aged 12–19 years from 1999 to 2020, using NHANES data [26]. This study found that the prevalence of prediabetes has tripled over the past 20 years, increasing from 11.5% in 1999–2002 to 36.3% in 2015–2020. This significant rise indicates a growing metabolic risk among adolescents in the United States over the last two decades. Obesity was identified as the major risk factor for both diabetes and prediabetes. Additionally, boys and Mexican American adolescents showed notably higher odds of developing prediabetes compared to their peers. The tripling of prediabetes prevalence underscores an urgent need for targeted prevention and intervention strategies, particularly aimed at obese youth and high-risk ethnic groups. The findings reinforce the importance of obesity prevention and early screening in adolescents.

#### 3.3.2. Family History of Type 2 Diabetes as a Non-Modifiable Risk Factor for Prediabetes and Type 2 Diabetes

The American Diabetes Association recognizes family history of type 2 diabetes as a key risk factor. Recent epidemiological studies and cohort analyses provide robust evidence quantifying its impact in pediatric populations [33]. Extensive systematic reviews and national data syntheses, such as the US Preventive Services Task Force evidence report, consistently identify family history of type 2 diabetes as a strong, independent predictor of both prediabetes and type 2 diabetes in children and adolescents, alongside obesity and pubertal stage [28,29]. These reviews highlight that family history remains a significant risk factor even after adjusting for other variables and is incorporated into validated risk assessment tools for pediatric diabetes screening.

Prospective cohort studies further clarify the magnitude of risk. For example, a multicenter study of overweight children found that a parental history of type 2 diabetes conferred an adjusted odds ratio of 6.3 to 9.5 for prediabetes, making it the strongest non-modifiable risk factor identified, surpassing even extreme obesity and pubertal status [34]. This association is supported by mechanistic studies demonstrating that children with a family history of diabetes exhibit early impairments in insulin sensitivity and secretion, even before the onset of clinical disease [35].

The American Diabetes Association states that early impairment of insulin sensitivity and insulin secretion can be detected in otherwise healthy children and adolescents who are offspring of individuals with type 2 diabetes [22]. This metabolic evidence of genetic susceptibility is often present in the first decade of life, even before overt hyperglycemia develops. The American Diabetes Association cites cross-sectional and longitudinal studies showing that youth with a family history of type 2 diabetes exhibit both reduced insulin sensitivity and impaired β-cell function compared to peers without such a history, and these defects precede the diagnostic thresholds for prediabetes and diabetes.

Recent analyses have also shown that the effect of family history is only partially explained by known genetic variants, suggesting additional contributions from rare genetic factors and shared environmental exposures [36]. The impact of biparental diabetes history is particularly notable, with hazard ratios for incident diabetes exceeding 1.4 in long-term follow-up studies, and maternal and paternal histories each independently increasing risk. These findings reinforce the importance of family history in risk stratification and early intervention strategies for pediatric populations. This highlights the importance of family history in risk stratification and prevention efforts.

#### 3.3.3. Gestational Diabetes, Prediabetes and Type 2 Diabetes Mellitus as Independent Risk Factors in the Offspring

The American Diabetes Association explicitly recognizes maternal history of gestational diabetes, prediabetes, and type 2 diabetes as important risk factors for the development of prediabetes and type 2 diabetes in offspring. The American Diabetes Association states that being the offspring of a pregnancy complicated by gestational diabetes mellitus or preexisting diabetes is a non-modifiable risk factor for youth-onset type 2 diabetes, supported by evidence from both animal and human studies [22]. In large cohort studies such as the SEARCH for Diabetes in Youth, intrauterine exposure to maternal gestational diabetes or diabetes and maternal obesity were independently associated with type 2 diabetes in adolescents, with nearly half of affected youth having such exposures [37].

The American Diabetes Association also highlights that maternal gestational diabetes confers a substantially increased lifetime risk of type 2 diabetes for the mother herself, and that this risk is further elevated in offspring, due to genetic, epigenetic, and intrauterine environmental mechanisms [38,39]. These guidelines cite studies showing that maternal gestational diabetes and diabetes are linked to early impairments in insulin sensitivity and secretion in children, which predispose them to prediabetes and type 2 diabetes later in life [40,41].

Building on the American Diabetes Association’s recognition of maternal diabetes as a risk factor, recent large-scale cohort studies and mechanistic research provide robust evidence that a maternal history of gestational diabetes, prediabetes, and type 2 diabetes directly increases the risk of prediabetes and type 2 diabetes in offspring through both genetic and intrauterine mechanisms [42,43]. The Hyperglycemia and Adverse Pregnancy Outcome (HAPO) follow-up study demonstrated that children exposed to gestational diabetes in utero have significantly higher rates of obesity and impaired glucose tolerance by early adolescence, with a continuous relationship between maternal glucose levels during pregnancy and offspring metabolic risk [44].

Systematic reviews confirm that all forms of maternal diabetes—gestational, preexisting type 2, and even milder forms of hyperglycemia—are associated with increased risk of obesity, glucose intolerance, and metabolic syndrome in offspring, with the highest risk observed in children of mothers with type 2 diabetes [45]. The findings are consistent across diverse populations and are supported by mechanistic data showing that maternal insulin resistance and dysglycemia during pregnancy program offspring for greater adiposity and impaired insulin secretion, independent of postnatal environment [44,46,47].

Notably, siblings born after a maternal diabetes diagnosis have a threefold higher risk of early-onset type 2 diabetes than those born before, underscoring the importance of the intrauterine environment. Intrauterine hyperglycemia acts through fetal hyperinsulinemia, impaired insulin sensitivity and secretion, and possible epigenetic changes to program offspring for increased risk of type 2 diabetes and prediabetes, beyond inherited genetic risk [48]. The increased risk persists even after accounting for family history of type 2 diabetes, highlighting the direct impact of the intrauterine environment [46]. Maternal hyperglycemia leads to increased fetal insulin secretion, which can alter pancreatic β-cell development and function. Offspring exposed to intrauterine hyperglycemia show reduced insulin sensitivity and β-cell dysfunction, as confirmed by follow-up studies in similar cohorts [49,50].

Exposure to high glucose in utero may induce lasting changes in gene expression and metabolic pathways, predisposing individuals to future dysglycemia [48,51,52]. Epigenetic studies further support that in utero exposure to maternal hyperglycemia leads to persistent changes in DNA methylation at loci associated with type 2 diabetes, contributing to transgenerational risk [47].

#### 3.3.4. Demographics and Ethnic Background

Multiple publications from the last 15 years demonstrate that ethnicity and demographics are significant risk factors for the development of prediabetes in children. Recent large-scale studies and systematic reviews consistently show that American Indian/Alaska Native, Black, Hispanic/Latino, and Asian/Pacific Islander youths have a higher prevalence of prediabetes compared to non-Hispanic White youths. For example, a 2025 cross-sectional study found that Asian and Pacific Islander adolescents with overweight or obesity had a markedly higher prevalence of prediabetes (26.9%) than non-Hispanic White adolescents (11.9%), with substantial variation among Asian subgroups (e.g., South Asian 31%, Filipino 28.2%, Chinese 25.9%) [53]. Similarly, a 2018 cohort study reported prediabetes rates of 55% in non-Hispanic Black, 43% in American Indian/Alaska Native, 37% in Hispanic, and 27% in non-Hispanic White adolescents, even after adjusting for BMI and socioeconomic factors [54]. National survey data and systematic reviews confirm these disparities. The US Preventive Services Task Force evidence report and recommendation statement highlight that the prevalence and incidence of prediabetes and type 2 diabetes are highest among American Indian/Alaska Native, Black, and Hispanic youth, and that these differences are likely influenced by both genetic and social determinants of health [28,29,55,56]. A 2024 NHANES-based study further demonstrated that Asian, Black, and Hispanic adolescents have higher prediabetes prevalence, and that adverse social determinants of health (SDOH)—such as food insecurity, public insurance, and low income—compound these risks across all racial and ethnic groups [57]. In summary, ethnicity and demographic factors—particularly race/ethnicity and socioeconomic status—are well-established risk factors for pediatric prediabetes.

Beyond statistical associations, several plausible mechanisms link race/ethnicity and SDOH to pediatric prediabetes. First, body composition differs across populations: at similar BMI, some groups, particularly Asian subgroups, exhibit greater visceral and hepatic fat, which confers higher insulin resistance and dysglycemia risk [58]. Hispanic/Latino youth have a high burden of hepatic steatosis, which is strongly associated with impaired insulin action [59]. Second, ancestry-related variation in beta-cell function and insulin sensitivity, especially during puberty, may predispose certain groups to earlier glycemic abnormalities [60]. Third, intergenerational influences, including higher rates of maternal obesity and gestational diabetes, can program offspring metabolic risk. Fourth, adverse SDOH—such as food insecurity, low income, and public insurance—operate through behavioral and physiologic pathways: they are linked to energy-dense diets, reduced opportunities for physical activity, chronic stress and sleep disruption, and constrained access to preventive care, all of which promote visceral adiposity and insulin resistance [61]. These mechanisms are consistent with the USPSTF evidence review’s conclusion that disparities likely reflect intertwined genetic susceptibility and social determinants [62]. The NHANES-based analysis showing compounding effects of adverse SDOH across all racial/ethnic groups supports this pathway perspective. Finally, differences in diagnostic modalities (e.g., HbA1c versus fasting glucose or OGTT) and known variation in HbA1c relative to glycemia across populations may influence case ascertainment; studies that adjust for SDOH and use multiple measures (as in the cited cohorts) strengthen confidence that the observed disparities reflect true differences in metabolic risk [63].

#### 3.3.5. Acanthosis Nigricans as a Prediabetes Risk Factor in Children

Acanthosis nigricans is a hyperpigmented, velvety thickening of the skin, most commonly found on the neck and axillae. Its presence in children, especially those with overweight or obesity, is strongly associated with underlying insulin resistance—a key pathophysiological driver of prediabetes and type 2 diabetes. Multiple guidelines, including those from the American Diabetes Association and the American Academy of Pediatrics, list acanthosis nigricans as a sign of insulin resistance and recommend that children with acanthosis nigricans and overweight/obesity be screened for prediabetes and type 2 diabetes [64,65,66]. Epidemiological studies confirm that children with acanthosis nigricans have a significantly higher likelihood of dysglycemia, even after adjusting for BMI and other risk factors and confounders [67,68].

The presence of acanthosis nigricans in children is a visible, easily recognized marker that identifies those at increased risk for prediabetes due to its strong association with insulin resistance. Its detection should prompt further evaluation for glucose abnormalities and early intervention [69,70,71].

#### 3.3.6. Polycystic Ovarian Syndrome

Polycystic ovarian syndrome (PCOS) is tightly linked to insulin resistance, which is present in up to 70% of affected adolescents, independent of obesity. This insulin resistance drives compensatory hyperinsulinemia, which not only exacerbates androgen excess and ovulatory dysfunction but also directly impairs glucose tolerance, increasing the risk for prediabetes and type 2 diabetes [72,73,74]. Epidemiological data show that adolescent girls with PCOS and obesity have an 18-fold higher incidence of type 2 diabetes compared to non-PCOS youth, with prediabetes often preceding diabetes by several years. Notably, even lean adolescents with PCOS demonstrate impaired glucose tolerance, underscoring that the risk is not solely attributable to excess adiposity [74,75,76]. The interplay between hyperinsulinemia and hyperandrogenism creates a vicious cycle, further reducing insulin sensitivity and promoting metabolic dysfunction [73,77].

Meta-analyses and cohort studies confirm that impaired glucose tolerance and prediabetes are common in adolescents with PCOS, with prevalence rates ranging from 10% to 45% depending on diagnostic criteria and population studied [76,78]. The risk of insulin resistance is amplified in those with additional risk factors such as Hispanic ethnicity, elevated HbA1c, and abnormal liver enzymes [79,80]. Furthermore, PCOS is associated with other cardiometabolic comorbidities, including dyslipidemia and hypertension, which compound the risk for future diabetes and CVD [17,81,82].

Adolescents with PCOS are at increased risk for abnormal glucose tolerance, including impaired glucose tolerance and type 2 diabetes mellitus, regardless of BMI, highlighting that factors beyond adiposity contribute to metabolic risk [76]. Gupta et al. found that insulin resistance and impaired glucose tolerance were more likely as BMI increased but occurred in both obese and non-obese youth [83]. In addition, since fasting plasma glucose and simple measures of insulin resistance are suboptimal for predicting impaired glucose tolerance and type 2 diabetes in this population, the 2-h plasma glucose level after a 75-g oral glucose tolerance test is the more reliable screening tool. PCOS confers a substantial and multifactorial risk for prediabetes in pediatric patients, mediated primarily by insulin resistance.

#### 3.3.7. Diet and Risk for Prediabetes

High intake of fructose, saturated fat, and overall poor dietary patterns are established risk factors for developing prediabetes in children. Excessive consumption of fructose, primarily from sugar-sweetened beverages and processed foods, promotes insulin resistance, hepatic fat accumulation, and dyslipidemia, all of which increase the risk of prediabetes and metabolic syndrome in youth [84,85]. Fructose metabolism in the liver drives de novo lipogenesis and uric acid production, contributing to obesity, fatty liver, and impaired glucose tolerance, even in normal-weight children. Reducing fructose intake improves insulin sensitivity, liver fat, and lipid profiles [86,87,88].

Diets high in saturated fat are associated with reduced insulin sensitivity and increased risk of prediabetes, especially in children with higher adiposity [89]. Longitudinal studies show that reducing saturated fat intake and increasing fruit and vegetable intake improve insulin sensitivity over time [90,91,92]. Saturated fat also promotes ectopic fat deposition and lipotoxicity, further impairing glucose metabolism [93].

The American Academy of Pediatrics and the Endocrine Society recommend dietary patterns emphasizing whole grains, vegetables, fruits, lean proteins, and unsaturated fats, while limiting processed foods, added sugars, and saturated fat [94,95]. Diets such as the Mediterranean diet and the Dietary Approaches to Stop Hypertension (DASH) diet (a heart-healthy eating plan designed to lower blood pressure) are associated with better glycemic and cardiometabolic outcomes [96,97]. Reducing sugar-sweetened beverages and ultra-processed foods is particularly effective for preventing obesity and prediabetes in children [98,99,100].

**Figure 1:**
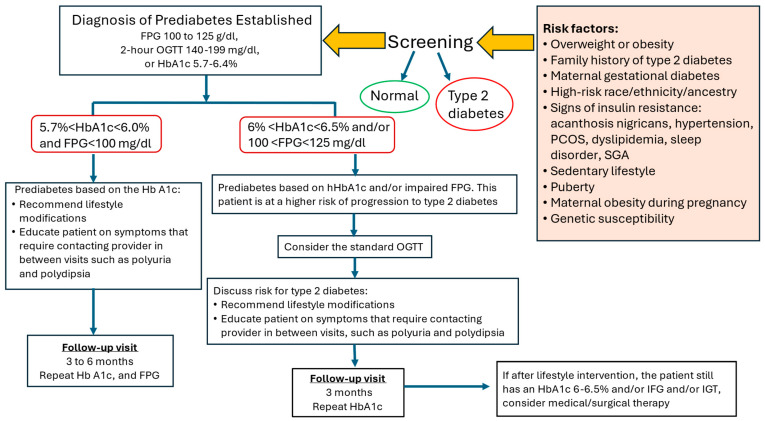
Clinical algorithm for pediatric patients with prediabetes. FPG: fasting plasma glucose; OGTT: oral glucose tolerance test; HbA1c: hemoglobin A1c; IFG: impaired fasting glucose; IGT: impaired glucose tolerance; PCOS: polycystic ovary syndrome; SGA: small for gestational age.

Figure 1 depicts a clinical algorithm summarizing the screening and treatment of prediabetes in children and adolescents. For children and adolescents, risk-based screening should be considered after puberty onset or age 10 years (whichever is earlier) in those with overweight (BMI ≥ 85th percentile) or obesity (BMI ≥ 95th percentile) plus one or more diabetes risk factors [101]. Following a diagnosis of prediabetes, intensive lifestyle modification is the first-line intervention, comprising a multidisciplinary approach to promote healthy lifestyle changes through education, nutrition counseling (ideally with an accredited dietitian), and structured physical activity programs (ideally with an exercise physiologist) [102]. Medical/surgical therapy should be considered for high-risk individuals.

## 4. Significant Physiological, Metabolic, and Biochemical Features of Prediabetes

### 4.1. The Continuum from Insulin Resistance to Type 2 Diabetes

The spectrum of dysglycemia ranges from insulin resistance and prediabetes to the full-blown disease state of type 2 diabetes [103,104,105]. Emerging literature suggests that the physiological changes associated with the disease, as well as related complications and risk factors, can occur even in the earlier stages of dysglycemia, before the development of type 2 diabetes [106,107,108,109,110]. With the rising prevalence of type 2 diabetes, there is a wealth of literature detailing the biochemical and physiological characteristics of the disease [111]. Additionally, there is a growing understanding of the associated risks related to microvascular and macrovascular complications linked to type 2 diabetes [112,113,114,115]. The inability of cells to process glucose leads to decreased triglyceride and glucose storage, ultimately causing hyperglycemia and hypertriglyceridemia. This progression results in type 2 diabetes and associated metabolic comorbidities such as hypertension, hyperlipidemia, and heart disease [116]. It should be noted that much of the data generated to date about the physiology and biochemical features of prediabetes has been performed in an adult cohort. Given the relatively recent rise in this state in the pediatric population, there is certainly a need to confirm these studies in a younger population. The authors will note when data is specific to pediatrics and when it is being generalized from known adult data.

### 4.2. Vascular Features of Prediabetes

Wallace et al. report evidence of both microvascular and cardiovascular changes in the prediabetic state in a study of 159,736 individuals [117]. Insulin resistance exacerbates oxidative stress and inflammation leading to nerve damage and apoptosis manifesting as retinopathy with visual impairment [118,119,120]. Not surprisingly, both adult and pediatric studies show retinopathy rates rise with poor glycemic control, diabetes and elevated blood pressure [121,122,123]. The incidence of retinopathy among adult patients with prediabetes is 8.2–20.9% [116,124]. A recent large multicenter adult cohort study revealed that risk factors associated with more frequent diabetic retinopathy included smoking, history of high blood pressure, elevated weight, hypertension, higher HbA1c, fasting and 2-h plasma glucose levels during an oral glucose tolerance test [125]. Though little pediatric data exists, pediatric studies show that diabetic retinopathy occurred with shorter diabetes duration in type 2 diabetes compared to type 1 diabetes, consistent with the broader literature showing more aggressive retinopathy development in youth-onset type 2 diabetes [123].

General nerve damage is a well-known consequence of diabetes that has also been found in the prediabetes cohort. Specifically, there is evidence of increased risk of distal sensorimotor polyneuropathy and cardiovascular autonomic neuropathy in persons with prediabetes [126,127,128,129]. A European study showed that 11–25% of people with prediabetes have peripheral neuropathy, and 13–21% of them have neuropathic pain [130]. The 2009–2014 NHANES Survey found that 7.5–16% of those with prediabetes had peripheral neuropathy [6]. A systematic review from Kirthi et al. found that peripheral neuropathy was present in more than 10% of those with pre-diabetes [131]. Though the above prediabetes studies are not pediatric studies, it is well known that the prevalence of peripheral neuropathy and autonomic neuropathy can impact about 11% and 15% of youth with type 2 diabetes, respectively [131]. A cohort study analysis from Jaiswal et al. found a prevalence of cardiovascular autonomic neuropathy in adolescents and young people below age 20 years in the United States with type 2 diabetes to be 17% [132]. Therefore development of neuropathy in earlier stages of prediabetes is an important consideration.

Similarly, heightened cardiovascular risk has been in the prediabetes population. The risk of stroke is increased in those adults with prediabetes compared to those without diabetes [116]. Based on data from the 2019 Behavioral Risk Factor Surveillance System survey from the state of Florida, Khan et al. found a stroke prevalence of 7.8% in adults with prediabetes versus 3.6% for the general population ibn Florida [133]. Welsh et al. used data from the UK Biobank to find a nearly two-fold higher unadjusted risk of CVD in individuals with prediabetes compared to those without diabetes [134]. Additionally, the prevalence of overall metabolic syndrome was 37.6% greater than that in normoglycemic patients. In a study from Lithuania, Smetania et al. reported that the odds ratio for prediabetes (fasting plasma glucose and 2-h postload glucose) and impaired glucose metabolism in association with metabolic syndrome among 10- to 17-year-old overweight and obese children and adolescents was 5.76 (*p* < 0.0001) and 3.44 (*p* < 0.0001), respectively [135]. Moreover, there are numerous studies supporting an increase in overall mortality and hospitalizations in those with prediabetes at baseline compared to those with normoglycemia [6].

### 4.3. The Kidney in Prediabetes

Kidney disease, which is so prevalent in type 2 diabetes, does not spare those with prediabetes [136,137]. The risk of kidney damage is already increased in the prediabetic state as the impact of hyperglycemia on the occurrence of impaired renal function may begin before glucose levels reach ranges consistent with diabetes mellitus [138]. In a 9-year prospective cohort study from Korea with over 7000 patients, Kim et al. showed in an adult cohort that meeting criteria for prediabetes was significantly associated with incident CKD after adjusting for traditional CKD risk factors (hazard ratio 1.4) [139]. A community-based large 7000-person cohort study of middle aged and elderly persons in China found a positive association between prediabetes and renal dysfunction as determined by estimated glomerular filtration rate [140].

### 4.4. The Brain in Prediabetes

Prediabetes is associated with cognitive issues related to both increased stroke risk and metabolic dysregulation [141,142]. Specifically, studies find that the risk of stroke increases as glycosylated hemoglobin rises [143,144]. Further, poor glucose control affects the brain structurally as shown by imaging studies. Grosu and colleagues looked at white matter hyperintensities, which are indicative of damage that may be hypoxia-induced or blood–brain barrier-related, in a German adult-aged cohort [145]. After adjusting for cardiovascular risk factors, they found a significant association between 2-h postprandial serum glucose and volume of white matter hyperintensities on MRI. Zhou et al. found a higher likelihood of white matter abnormalities in adults with prediabetes [146]. Jing et al. also detected microstructural abnormalities in white matter in prediabetes [147]. Multimodal neuroimaging from Chen et al. found cerebellar damage in pre-diabetes that worsened with diabetes [148].

Multiple studies show that prediabetes is associated with mild cognitive impairment [149,150]. Sundermann et al. measured cerebral metabolic glucose rate and found it to be lower in prediabetic patients than in those with normoglycemia. However, prediabetes was associated with a decline in executive function only in adult women [151]. A major nationwide cohort study from Korea found that greater cumulative exposure to impaired fasting glucose (defined as fasting glucose between 100 and 125 mg/dL) increased the risk of all-cause dementia or Alzheimer’s disease in the non-obese group, but not in the obese group [152]. Though pediatric data is very limited, the link between hyperglycemia and cognitive ability is notable, warranting further pediatric studies.

### 4.5. Physiological Features of Prediabetes

Physiologically, insulin resistance and prediabetes exist on a continuum with shared pathogenic processes. Insulin resistance impacts both hepatic and peripheral tissues alike. Resistance at the level of the liver leads to both increased endogenous glucose production and decreased glucose clearance. There is also thought to be a compromise of the glucose feedback mechanism interfering with its ability to increase glucose uptake and suppress hepatic production [6,153]. Those with the beginning stages of insulin resistance and impaired glucose tolerance show additional resistance at the level of the skeletal muscle that leads to defects in insulin signaling that impedes glucose uptake and raises blood glucose. This, in turn, leads to β-cell dysfunction in the pancreas which delays glucose uptake and causes blood glucose to rise even more [154,155]. In addition to reduced hepatic insulin sensitivity, β-cell dysfunction, and some evidence of low β-cell mass, there are also studies showing altered glucagon-like peptide-1 secretion and inappropriately elevated glucagon secretion among the multiple factors leading to a state of prediabetes [156,157,158]. Interestingly, there is also a noted heterogeneity in the pathophysiology of prediabetes in that. some models indicate inherent β-cell dysfunction leading to worsening resistance and risk for diabetes, while other models show insulin resistance as the first pathophysiologic mechanism that then causes further β-cell dysfunction [159,160,161]. This may indicate that type 2 diabetes cannot be thought of as one single disease with a linear pathophysiology but may represent a more complex overlap of disease states and disease development [162].

### 4.6. Biochemical Features of Prediabetes

There is an evolving literature about the pathophysiology of prediabetes from a cellular and biochemical perspective. People with prediabetes, including the pediatric population, tend to have decreased suppression of glucagon and disproportionately elevated glucagon levels, leading to α-cell dysfunction in addition to the β-cell dysfunction already described [157,163,164]. There remains a debate over the biochemical contribution of GLP1 levels in the pathogenesis of prediabetes. Some studies have shown impairment, while others have not [165,166,167,168,169].

On a cellular level, in a healthy state, as blood sugars rise postprandially, levels of AMP-activated protein kinase (AMPK), a sensor of the energy state, decline within the islet cells, triggering activation of AMPK phosphorylation and enhancing insulin secretion [170]. This activates muscle-induced glucose uptake and decreases hepatic gluconeogenesis. In damaged pancreatic β-cells, a sustained high-glucose environment results in AMPK phosphorylation in the pancreatic β-cells, inhibiting glucose-stimulating insulin secretion and promoting insulin resistance [171]. Moreover, there is evidence that the pre-diabetic state induced endoplasmic reticulum stress and MTOR activation, leading to deterioration of β-cells and further apoptosis [172,173,174]. During the progression towards type 2 diabetes, there is a chronic activation of the Mammalian Target of Rapamycin Complex 1 (mTOR) complex 1 signaling pathway, which induces aging and serves as an endogenous inhibitor of autophagy. The mTOR complex 1 pathway regulates cell proliferation, growth, and metabolism in various cell types through a complex signaling network. Autophagy plays a crucial role in recycling cellular components to generate energy during nutrient deprivation and acts as an alternative degradation system to the ubiquitin-proteasome pathway [175]. Additionally, autophagy serves as a protective mechanism for different cell types, including pancreatic β-cells, and enhances β-cell survival during the progression to type 2 diabetes [116,176]. The loss of β-cell mass and function results in insulin resistance and β-cell apoptosis secondary to insufficient insulin secretion then begets a further decrease in glucose uptake and increasing hyperglycemia which then leads to further hyperglycemia in a cyclic fashion [177]. In short, the physiology and metabolic risk profile of diabetes certainly starts early in the prediabetic state, giving credence to the need for attention to this condition well before it reaches Type 2 diabetes.

## 5. Lipolysis, Incretin, Alpha Cell, and Inflammation in Prediabetes

### 5.1. Lipolysis

Childhood obesity is considered a significant risk factor for the development of prediabetes in children [178]. Typically, there is a balance between lipid storage and breakdown. Lipogenesis in the liver and adipocytes yields triglycerides as a form of energy storage. Lipolysis, an enzymatic breakdown of triglycerides stored in adipose tissue into free fatty acids and glycerol, releases fatty acids for energy. Insulin secreted by β-cells of the pancreas promotes uptake of glucose and free fatty acids and reduces lipolysis by inhibiting the key lipolytic enzyme lipase.

**Figure 2:**
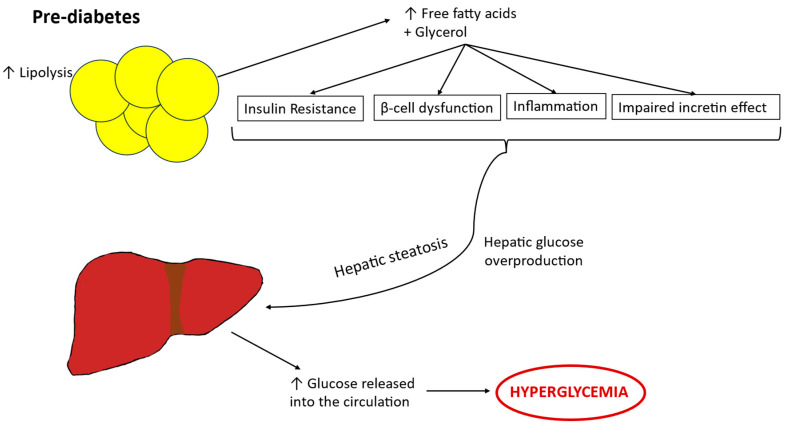
Increased lipolysis is associated with a significant rise in free fatty acids and glycerol in the serum, which contributes to insulin resistance, β-cell dysfunction, inflammation, and an impaired incretin effect, ultimately leading to hepatic glucose overproduction. ↑ = increased.

Prediabetes is characterized by increased lipolysis, which incites hepatic glucose overproduction, insulin resistance, β-cell dysfunction, inflammation, and impaired incretin effect [179,180]. Between 80% and 90% of the mass of the adipocyte is triglyceride [181]. As a result of lipolysis there is a significant increase in free fatty acids in the serum of obese subjects [182]. The overflow of these free fatty acids in obese serum is responsible for obesity-associated insulin resistance, beta cell toxicity and hepatosteatosis which further highlight the relationships among lipid metabolism, insulin secretion and insulin sensitivity [183,184,185] (Figure 2). Kim et al. demonstrated that youth with impaired glucose tolerance exhibited increased lipolysis, β-cells dysfunction and decreased hepatic and peripheral insulin sensitivity which raise the risk of developing type 2 diabetes [185].

### 5.2. Incretins

Incretins are the hormones secreted from the gastrointestinal cells in response to food intake. Incretins stimulate glucose-induced insulin secretion from the pancreas, thus regulating blood glucose levels after meals [186]. The two main incretin hormones are GLP-1 (Glucagon-like peptide-1) and GIP (Glucose-dependent insulinotropic polypeptide). Apart from increasing insulin secretion, incretins also suppress the secretion of glucagon [187,188]. Studies have shown that the levels of circulating incretins are altered in patients with prediabetes [189]. Abnormal incretin secretion contributes to the dysfunction of β-cells in prediabetes and type 2 diabetes [190]. Following an oral glucose challenge, GLP-1 levels are reduced in individuals with impaired glucose tolerance and prediabetes [168]. Moreover, these individuals exhibit impaired glucagon suppression [191].

Due to the beneficial effects on weight loss and metabolic parameters, the use of incretins in patients with prediabetes is considered to have benefits [192]. In addition to improving β-cell function and glycemic control, incretin-based therapy has favorable effects on weight loss, the liver, and the cardiovascular system [191,193,194].

Despite these beneficial effects, in clinical practice, the primary treatment approach for pediatric prediabetes remains lifestyle interventions focused on weight loss, improved diet, and increased physical activity. Incretin-based therapies, particularly GLP-1 receptor agonists, have become clinically relevant for pediatric practice as effective pharmacologic options for treating obesity and type 2 diabetes in children and adolescents, with several agents now FDA-approved for use in patients as young as 6–10 years of age. For weight management, these agents produce mean weight reductions of 2.89–3.02 kg and decrease BMI by approximately 1.45 units, with BMI standard deviation scores improving by 0.20 [132]. The American Diabetes Association now includes GLP-1 receptor agonists in treatment algorithms for youth with type 2 diabetes, noting their safety and effectiveness for both glycemic control and weight loss [195]. GLP-1 receptor agonists are not FDA-approved for prediabetes and are only indicated for patients with both prediabetes and obesity.

### 5.3. Alpha Cells

Many studies have focused on the pathophysiology of prediabetes and β-cell dysfunction, but the role of alpha cells has received less attention [196]. In response to variety of nutrients, hormonal factors and hypoglycemia, glucagon is released from alpha cells [197,198]. Insulin and glucagon actions are counterregulatory. Insulin lowers blood glucose levels by increasing the uptake of glucose in peripheral tissue, whereas glucagon increases blood glucose levels by increasing the hepatic glucose production [199,200]. The excessive release of glucagon from alpha cells of the pancreas causes hyperglycemia in type 2 diabetes [201,202]. Chang et al. described the insufficient suppression of glucagon levels in individuals with prediabetes in comparison to individuals with normal glucose tolerance during the oral glucose tolerance test (OGTT), suggesting that individuals with prediabetes exhibited higher absolute glucagon levels during OGTT compared to those with normal glucose tolerance [157]. Roncero-Ramos et al. used data from the CORDIOPREV study, a clinical trial from Spain in which patients were randomized to either a Mediterranean diet or a low-fat diet intervention to show that glucose levels in the OGTT measured at baseline and after 2 years were higher at baseline in those who went on to develop type 2 diabetes than in those who went on to develop pre-diabetes [203,204]. The glucose levels on OGTT did not differ between those who went on to develop pre-diabetes and controls. Glucagon levels followed this same pattern. From these findings, they infer that alpha cell dysfunction occurs prior to the development of type 2 diabetes. A study of children between the ages of 7 and 17 years with obesity and insulin resistance from Stinson et al. found that fasting concentrations of glucagon and GLP-1 were elevated compared to normal weight controls [205]. During OGTT, the group with obesity and insulin resistance showed an attenuated GLP-1 response.

Blocking the glucagon receptor or inhibiting glucagon secretion are considered as a promising pharmacological target for antidiabetic treatment [206]. Some of the glucagon receptor antagonists are glucagon-derived peptides, non-peptide small molecules, monoclonal antibodies, and antisense oligonucleotide molecules [207]. Incretin mimetics, particularly GLP-1 analogues, offer promising advantages for managing HbA1C levels and regulating serum glucagon levels. Their use can be an effective strategy in improving overall glycemic control [208,209,210].

### 5.4. Inflammation and Prediabetes

Prospective studies have shown that chronic low-grade inflammation often occurs before the onset of diabetes [211]. Elevated levels of certain biomarkers, including C-reactive protein, white blood cell count, IL-1β, interleukin-1 receptor antagonist (IL-1RA), IL-6, IL-8, IL-18, MCP-1, interferon-γ-inducible protein-10 (IP-10), haptoglobin, and fibrinogen, indicate a persistent, often subclinical level of inflammation. These biomarkers can be elevated years before an individual develops type 2 diabetes [212]. Furthermore, the level of subclinical inflammation has been reported to be similar in individuals with impaired fasting glucose and those with diabetes [213]. Chen et al. found that a medium or high score on The Children’s Dietary Inflammatory Index, a measure of systemic inflammation, was associated with a 20% increase in risk for prediabetes in adolescents [214,215].

Adipose tissue was once thought to be just a passive reservoir for energy storage [216]. However, it is now recognized as a significant endocrine organ that secretes adipokines, cytokines, and chemokines. Inflammation of adipose tissue may play a central role in the development of insulin resistance associated with obesity, contributing to various underlying mechanisms involved in this condition [217]. The recognition of adipose tissue as an endocrine organ showed that some secreted hormones, such as adiponectin, could affect systemic insulin sensitivity [218].

**Figure 3:**
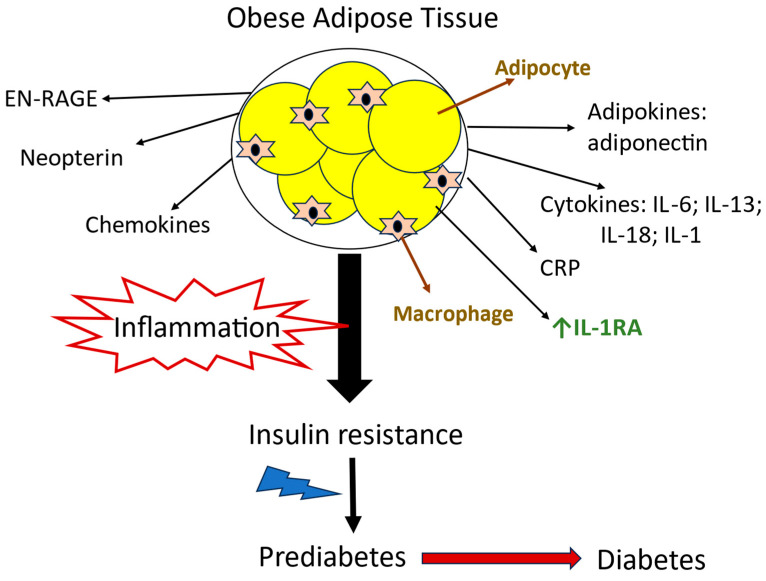
The secretory function of adipocytes connects adipose tissue, inflammation, and prediabetes. Inflammation within adipose tissue is likely a key factor in the development of insulin resistance that occurs with obesity. Increased levels of pro-inflammatory markers, such as EN-RAGE, interleukin (IL)-6, IL-13, IL-18, IL-1, C- reactive protein (CRP), have been linked to the development of insulin resistance, prediabetes, and type 2 diabetes mellitus. The IL-1 receptor antagonist (IL-1RA) is anti-inflammatory mediator produced by adipose tissue that rises in prediabetes. ↑IL-1RA = increased IL-1RA.

Several pro-inflammatory and anti-inflammatory biomarkers such as adiponectin, extracellular newly identified-RAGE (EN-RAGE), IL-6, IL-13, CRP, IL-18, IL-1 receptor antagonist, and neopterin have been associated with the progression from prediabetes to diabetes. EN-RAGE is a calcium-binding protein that promotes inflammation [219]. Its most well-known targets are RAGE (Receptor for Advanced Glycation Endproducts) and TLR4 (Toll-like receptor 4) [220]. Both RAGE and TLR4 serve as gatekeepers of the innate immune system and trigger inflammatory cascades that involve pathways such as nuclear factor (NF)-κB and c-Jun NH2-terminal kinase (JNK). These pathways play significant roles in the development of insulin resistance and type 2 diabetes mellitus [221]. Adiponectin, a hormone derived from adipose tissue, is crucial for regulating glucose levels and lipid metabolism [222]. Low level of adiponectin contribute to inflammation and decreased insulin sensitivity in prediabetic patients during hyperglycemia [223]. The secretory nature of adipocytes links adipose tissue, inflammation, and prediabetes [218,224] (Figure 3). Studies show increased levels of both pro-inflammatory and anti-inflammatory markers, such as IL-1RA, TGF-β1, and growth differentiation factor (GDF)-15 in patients with prediabetes [212,225]. Elevated plasma glucose is one of the factors involved in causing chronic inflammation in prediabetes while elevated anti-inflammatory markers in patients with prediabetes might be an attempt to counteract increased proinflammatory activity in the body [226].

## 6. Pediatric CVD as a Complication of Prediabetes

### 6.1. Pathophysiology of CVD and Underlying Processes Beginning in Childhood

CVD is the leading cause of death and disability in industrialized countries and brings tremendous healthcare costs [227,228,229]. While CVD typically occurs in adulthood, damage to the cardiovascular system, such as arterial stiffening, can begin early in life. Alterations that promote the formation of atherosclerotic plaques may already be present in children as early as the first decade of life [230].

The evidence linking prediabetes itself to cardiovascular changes in children, independent of obesity, is limited and methodologically weak [178,231,232,233]. Most studies show associations between prediabetes and cardiovascular risk factors in youth, but these relationships are largely confounded by obesity, making it difficult to isolate the independent effect of prediabetes. The fundamental limitation is that nearly all pediatric studies of prediabetes include predominantly or exclusively youth with obesity, reflecting the strong co-occurrence of these conditions [234]. Without adequate representation of normal-weight youth with prediabetes, isolating the independent cardiovascular effects of dysglycemia remains challenging [235].

The early onset of atherosclerosis is a concerning issue, as demonstrated by findings from the Bogalusa Heart Study conducted several years ago [236]. Post-mortem biopsy studies of children aged 2 to 15 revealed that nearly all these children had fatty streaks in their aorta, and about 50% had fatty streaks in their coronary arteries. Furthermore, the prevalence of fibrous plaques was approximately 20% in the aorta and 8% in the coronary arteries. The severity of these lesions in both the aorta and coronary arteries was strongly associated with higher BMI, elevated blood pressure, and changes in lipid profiles. This information is particularly alarming given the significant rise in childhood obesity over the past few decades. A recent study utilizing NHANES data, which included 25,847 participants, found that the prevalence of extremely severe pediatric obesity increased from 0.32% in 2008 to 1.13% in 2023 [237]. The study also demonstrated that children with extremely severe obesity faced a higher risk of developing conditions such as metabolic dysfunction-associated steatotic liver disease (MASLD), prediabetes or diabetes, severe insulin resistance, and metabolic syndrome when compared to those with obesity classes 1 to 3. Children and adolescents with obesity have a higher prevalence of abnormal cholesterol levels compared to those of normal weight or who are overweight [238]. Furthermore, childhood obesity and metabolic syndrome can cause dyslipidemia and early vascular disease [239,240,241]. Approximately 1 in 5 children and adolescents in the United States, aged 6–19, have at least one abnormal cholesterol measure, including high total cholesterol, low high-density lipoprotein (HDL) cholesterol, or high non-HDL cholesterol [242].

Vascular lesions that develop at a young age and lead to atherosclerosis primarily occur in the sections of the arterial tree where blood flow is turbulent [243,244]. Hemodynamic forces exerted on the endothelial cells affect their permeability and modify the expression of specific endothelial cell genes, such as that for nitric oxide synthase [245]. As a result, this alters the production of nitric oxide. This situation promotes the infiltration of low-density lipoprotein (LDL) into the arterial wall, where they undergo oxidation. This oxidation leads to an inflammatory state that attracts monocytes, which further trigger other inflammatory factors, creating a vicious cycle. The monocytes eventually transform into macrophages. As these macrophages accumulate excess lipids, they become foam cells, marking the genesis of atherosclerotic plaque [246]. It has become apparent that preventive measures should begin in childhood [247].

### 6.2. Biomarkers of CVD in the Pediatric Population

Cardiovascular risk factors in childhood are linked to the development and severity of vascular dysfunction, as well as both subclinical and manifest atherosclerosis in children and adults [248,249]. Circulating biomarkers, including high serum glucose and lipid levels, as well as inflammatory markers and the systemic effects of high blood pressure, adversely affect the vasculature, leading to damage in both the macro- and microcirculation of target organs [249,250]. Identifying children at risk of developing CVD as early as possible allows for the implementation and maintenance of optimal health behaviors when they are most likely to be effective. The availability of metabolic screenings presents an opportunity to identify biomarkers associated with cardiovascular and metabolic risk [251].

Previous studies have demonstrated that obesity, hypertension, insulin resistance, dyslipidemia, type 2 diabetes, and CVD are associated with multiple circulating metabolites such as branched-chain and aromatic amino acids in adults [252,253,254,255,256]. CVD biomarkers such as glycoprotein acetyls (GlycA), very large high-density lipoprotein phospholipids (L-HDL-PL), and the apolipoprotein (Apo)B to ApoA1 ratio are significant predictors of future cardio-metabolic health in childhood and adolescence [257].

A significant inverse relationship between childhood L-HDL-PL and cardiometabolic risk during early adulthood has been identified, suggesting that exposure to an atherogenic apolipoprotein profile and low HDL-PL in childhood may lead to reduced cholesterol efflux capacity and changes in the arteries that contribute to the development of atherosclerosis and coronary heart disease in adulthood [258]. Previous research has shown that HDL phospholipids play a significant role in the cholesterol efflux process [259]. Moreover, metabolic syndrome is linked to a gradual decline in cholesterol efflux capacity, which plays a role in the progression of atherosclerosis [260,261].

Longitudinal data from Finland has shown that the serum ratio of ApoB/ApoA1 in childhood is a strong and reliable predicter of future cardio-metabolic risk score. ApoB and ApoA1 are the two main lipoproteins involved in lipid transport. ApoB is the primary protein found in atherogenic lipoprotein particles, such as very-low-density lipoproteins (VLDL) and LDL. According to the 2019 guidelines from the European Society of Cardiology and the European Atherosclerosis Society, measuring ApoB provides a more precise evaluation of cardiovascular risk, and the effectiveness of lipid-lowering treatments compared to measuring LDL cholesterol (LDL-c) or non-high-density lipoprotein cholesterol (non-HDL-c). Additionally, ApoB measurement is remarkably accurate at lower concentrations compared to LDL-c or non-HDL-c [262,263]. ApoA1 is the main protein in the atheroprotective lipoprotein HDL and plays a key role in reverse cholesterol transport and cellular cholesterol homeostasis [264,265]. In a prospective study conducted by Juonala et al., levels of ApoB and ApoA1, as well as their ratio, during adolescence were linked to carotid artery intima-media thickness (IMT) and brachial artery flow-mediated dilation in adulthood [266]. Their findings indicated that ApoB and ApoA1 were stronger predictors of abnormal vascular changes than conventional cholesterol measurements, such as LDL-C and HDL-C. This suggests that the apolipoproteins may play a more significant role than the actual lipid content transported within these lipoprotein particles. These findings are further supported by the Bogalusa Heart Study, which demonstrated that a high ApoB/ApoA1 ratio in children is linked to the incidence of parental myocardial infarction [267]. A systematic review and meta-analysis from Dinpanah et al. brings in further concordant results showing that an elevated ApoB/ApoA1 ratio is associated with metabolic syndrome in children and adolescents [268]. In addition, a recent study also showed that an increased ApoB/ApoA1 ratio predicts cardiometabolic risk in patients with juvenile-onset systemic lupus erythematosus [269].

It is increasingly recognized that the atherosclerotic process involves not only the metabolism of lipids and lipoproteins but also requires a pro-inflammatory response that engages both the innate and adaptive immune systems [270,271]. Recent studies, along with earlier reports, have shown that GlycA holds promise as a viable biomarker for systemic subclinical inflammation [272]. GlycA reflects systemic inflammation due to glycan groups from acute-phase glycoproteins, primarily a1-acid glycoprotein, as well as other acute-phase reactants like haptoglobin, alpha1-antitrypsin, alpha1-antichymotrypsin, and transferrin. This inflammation is linked to an increased risk of cardio-metabolic issues in both adults and youth, including children and adolescents [258,273,274].

### 6.3. Modifiable CVD Risk Factors in Children and Adolescents

The National Institutes of Health (NIH) has classified the risk factors for CVD into two main categories: non-modifiable and modifiable factors [275]. Non-modifiable risk factors include age, sex, race/ethnicity, family history, genetic predispositions, congenital conditions, and socioeconomic status. Modifiable risk factors can be further divided into two subgroups: 1. Cardiometabolic factors, which include hypertension, diabetes, and dyslipidemia. 2. Lifestyle factors: Encompassing physical inactivity, diet, and obesity [276]. Studies on adults have indicated that, compared to normoglycemia, prediabetes is linked to an increased risk of atherosclerotic CVD and overall mortality [277]. In addition, adolescents who develop type 2 diabetes are known to have a more rapid decline in glycemic control and progression of complications [278,279]. Diabetes can lead to both microvascular and macrovascular complications. These complications include issues such as microalbuminuria, hypertension, and dyslipidemia [280,281]. The Bogalusa Heart Study showed that LDL-C levels and BMI measured during childhood could predict carotid IMT in young adults [282]. Therefore, addressing cardiometabolic risk factors and lifestyle factors in childhood and adolescence may help prevent atherosclerosis in adulthood.

One of the main reasons for diagnosing prediabetes is to identify individuals at risk of complications associated with type 2 diabetes mellitus and CVD [14,283]. The risk of developing CVD is a continuum that gradually increases based on risk factors, which can accumulate and combine, progressively leading to endothelial damage, vascular and myocardial remodeling, and atherosclerotic processes [284]. Addressing the modifiable risk factors present during childhood and adolescence can help prevent atherosclerosis in adulthood [285]. Table 2 provides an overview of both the modifiable and non-modifiable CVD risk factors in the pediatric population. The modifiable factors could translate into routine pediatric screening or risk stratification for patients with prediabetes.

Atherosclerosis usually begins in childhood as preclinical atherosclerosis and develops quietly for decades, typically affecting middle-aged and older adults before leading to clinical events like stroke, myocardial infarction, and peripheral vascular disease [286,287]. Preclinical atherosclerosis refers to the initial stages of atherosclerotic disease. During this phase, structural and functional changes occur in the arterial walls, such as thickening, the accumulation of coronary calcium deposits, and increased rigidity [288,289]. These changes affect the elasticity and reactivity of the arteries, leading to abnormal thrombogenicity, all without causing any noticeable symptoms. This stage is critical for early detection and intervention to prevent the progression to symptomatic cardiovascular disease [290,291]. Therefore, it is essential to ensure the early detection of preclinical atherosclerosis and related risk factors, as they are major predictors of cardiovascular disease and stroke later in life.

Common carotid artery IMT assessed by ultrasonography is a well-established marker of subclinical atherosclerosis [292,293]. The Bogalusa Heart Study demonstrated that childhood levels of LDL-C and BMI can predict carotid IMT in young adults [294]. The Cardiovascular Risk in Young Finns Study found that LDL-C, systolic blood pressure, BMI, and smoking habits in adolescents aged 12–18 years are significant predictors of IMT in young adults aged 33–39 years [295].

Obesity, the most common cause of insulin resistance in children, is also associated with dyslipidemia type 2 diabetes, and long-term vascular complications [296,297,298,299,300,301]. Pediatric obesity is classified as follows: traditional classes 1 to 3 obesity (BMI ≥ 95th percentile to <160% of the 95th percentile) and extremely severe obesity, comprising class 4 (BMI ≥ 160% to <180% of 95th percentile) and class 5 (BMI ≥ 180% of 95th percentile) obesity [17,237]. Research continues to highlight a strong association between obesity and metabolic syndrome in adolescents [302]. Studies show that obesity, particularly severe obesity, is linked to a higher risk of metabolic complications like MASLD, prediabetes, and metabolic syndrome itself [303,304]. The metabolic syndrome is a collection of risk factors that increase the likelihood of developing heart disease, stroke, and diabetes in the pediatric and adult population [299,305]. Features of insulin resistance and metabolic syndrome include upper-body obesity, abnormal glucose metabolism, hypertriglyceridemia, decreased HDL-C levels, and hypertension [306,307]. Components of the metabolic syndrome can often be identified in prediabetic individuals several years before a diagnosis of type 2 diabetes mellitus [308,309]. In a sample of adolescents in the United States included in the third National Health and Nutrition Examination Survey (NHANES III), conducted between 2008 and 2023, the prevalence of the metabolic syndrome was 4.44% for participants without obesity, 36.91% for those with obesity classes 1 to 3, and 53.75% for those with obesity classes 4 to 5 [237]. In addition, this study revealed that the prevalence of prediabetes or type 2 diabetes was significantly higher among participants with obesity classes 4 to 5 (46.77%) than among those with classes 1 to 3 (15.10%) and those without obesity (5.99%).

Childhood and adolescent obesity are associated with several well-known risk factors for CVD and can lead to the development of atherosclerosis [310,311]. These risk factors include elevated blood pressure, atherogenic dyslipidemia, metabolic syndrome, type II diabetes mellitus, changes in cardiac structure and function, and obstructive sleep apnea [312,313]. Obesity triggers chronic, low-level inflammation in fat tissue, particularly in visceral fat, which is linked to an increased risk of CVD [314,315]. Excess weight that is metabolically unhealthy is characterized by an increase in visceral fat, a decrease in subcutaneous fat, enlarged fat cells (known as adipocyte hypertrophy), elevated secretion of inflammatory factors, and the accumulation of fat in inappropriate areas (referred to as ectopic fat deposition) [300,316]. Young individuals with excess visceral fat often exhibit significantly higher levels of atherogenic lipoproteins and are more likely to experience dysglycemia [316,317,318,319].

The relationship between childhood obesity and its metabolic complications on the progression of atherosclerosis is still being studied [306,320]. This progression involves four stages: endothelial dysfunction, lipid accumulation, plaque formation, and plaque rupture [321]. Both cross-sectional and longitudinal studies of youth cohorts have indicated that obesity, with or without type 2 diabetes, may lead to an accelerated rate of vascular aging. This refers to the deterioration of vascular structure and function among young people and young adults [308,322].

Research in adults has indicated that the metabolic syndrome and prediabetes often identify similar populations. However, the metabolic syndrome is more likely to detect individuals with early signs of renal dysfunction and increased inflammatory activity. At the same time, prediabetes tends to identify those with early structural changes in the carotid arteries [323,324]. A study conducted by Diamantopoulos et al. found that metabolic syndrome is associated with a higher body BMI, elevated serum triglyceride levels, a higher triglycerides-to-HDL cholesterol ratio, and increased non-HDL cholesterol levels. In contrast, prediabetes is associated with lower HDL cholesterol levels, a lower HDL-to-LDL cholesterol ratio, and higher fasting blood glucose levels [323]. The study also indicated that individuals with prediabetes had a significantly lower acute-phase insulin response, as measured by the insulinogenic index, and higher insulin resistance, marked by the HOMA-IR score. Multiple studies have demonstrated that increased carotid IMT, which indicates the presence of early atheromatous vascular lesions, is observed in individuals with impaired glucose tolerance or those with concurrent cardiovascular disease risk factors [324]. Bulut et al. also found that higher carotid IMT values are linked to prediabetes and insulin resistance [325]. However, this association diminishes when considering the other components of metabolic syndrome. These differences may arise from the distinct underlying pathophysiology of these clinical conditions, particularly in relation to insulin resistance and secretion, or from variations in the prevalence of other cardiovascular risk factors [326,327].

## 7. Developing Therapeutic Approached

### 7.1. Managing Obesity

Obesity is a significant risk factor for developing prediabetes. The two conditions are closely connected [328,329]. Excess adiposity leads to insulin resistance, dyslipidemia, hypertension, and chronic inflammation—all of which contribute to the pathogenesis of prediabetes, type 2 diabetes, and cardiovascular disease [330]. Therefore, treatment of obesity is an essential step in preventing or reversing prediabetes or delaying the onset of type 2 diabetes. Various interventions, including lifestyle modifications, pharmacotherapy, and even metabolic bariatric surgery, can help reduce weight, thereby improving insulin sensitivity and glycemic control.

### 7.2. Lifestyle Interventions

The foundation of treatment for childhood obesity is lifestyle modifications focused on diet, exercise, and behavioral therapy [331]. The Obesity Working Group of the Latin American Society for Pediatric Gastroenterology, Hepatology, and Nutrition (LASPGHAN), as reported by Rivera Suazo et al., recommends avoiding added sugar for children under the age of 2 years [332]. For children aged 2 to 18 years, LASPGHAN recommends limiting added sugar to less than 5% of daily energy intake. Additionally, they advised that children engage in 60 min of exercise daily. However, benefits for cognitive and metabolic health have been observed even with just 20 min of exercise, 3 to 5 times per week.

Motivational interviewing, developed by psychologists William R. Miller and Stephen Rollnick, is a collaborative, person-centered approach to helping individuals strengthen their own motivation and commitment to change [333]. The efficacy of motivational interviewing has been uncertain [334,335]. In a 2015 study, Resnicow et al. found that when providers and Registered Dietitians delivered motivational interviews, it led to statistically significant reductions in BMI percentile [336]. However, a more recent study from Resnicow failed to show a benefit from motivational interviewing [337].

Endocrine Society Clinical Practice Guidelines recommend pharmacotherapy for children and adolescents younger than 16 years of age with obesity only after a formal program of intensive lifestyle modification has failed to lead to weight loss. Moreover, children whose BMI percentile is in the overweight range, but not the obese range, should not be offered weight loss medications, which so many patients are looking for now [95,338]. Even if pharmacotherapy is started, it must be accompanied by lifestyle intervention focused on diet, exercise, and behavioral therapy [331,339].

Interventions that effectively improve BMI usually involve the participation of a nutritionist, alongside physical activity and a behavioral treatment approach, particularly if there is less than 26 h of contact time over 2 to 12 months [340]. Interventions that include 26 h or more of contact time often incorporate actual physical activity training during visits and provide behavioral health support. The most successful interventions combined elements of nutrition and exercise, as well as peer support groups, and were delivered in person [341,342]. These were recommendations consistent with the United States Preventive Services Task Force (USPSTF) [343]. However, even in ideal research conditions, it was challenging to deliver this high number of contact hours.

Individualized programs with lifestyle interventions tailored to the child and family and including parental involvement are gaining support [344]. Dietary and lifestyle interventions, specifically outlined by Lauren Williams, RDN, for youth with dyslipidemia and hypertension, focus on targeted strategies [345]. For dyslipidemia, the Williams paper highlights reducing saturated and trans fats, increasing soluble fiber intake (6 g/day for ages 2–12 and 12 g/day for older youth), and limiting sugar-sweetened beverages to help control triglyceride levels and obesity. Foods high in soluble fiber include flaxseeds, beans, avocados, and oats. For hypertension, recommended lifestyle changes comprise regular physical activity, sodium reduction, weight management, and a diet rich in fruits, vegetables, low-fat dairy, and whole grains. Engaging family and using motivational interviewing are employed to maintain these changes. Robins et al. describe that parent-involved physical activity and nutrition interventions have the potential to lower BMI and enhance other health outcomes related to weight [346].

Telehealth and digital tools may be useful in supporting weight loss in youth, but more studies are needed. Struckmeyer et al. described a retrospective study evaluating the long-term effects of a structured telehealth lifestyle intervention for pediatric obesity compared to historical in-person care [347]. They conducted a study at a single academic center that included 237 children and adolescents with obesity—117 patients were treated via telehealth from 2020 to 2022, and 120 had an in-person visit from 2017 to 2019. Both groups participated in an identical 12-month lifestyle program, with the telehealth group followed for up to 36 months. The primary outcome measure was change in BMI standard deviation score (BMI SDS), while secondary outcomes included physical fitness (6-min walk test), insulin resistance (HOMA index), lipid profile, dietary habits, eating regulation, and health-related quality of life. Both groups experienced significant reductions in BMI SDS after 12 months; additionally, the telehealth group maintained these improvements for up to 36 months (Δ = −0.18; *p* < 0.05). Gains in physical performance and insulin sensitivity were comparable across groups, but the telehealth group showed greater progress in healthy dietary habits, cognitive appetite regulation, and quality of life. Adherence rates exceeded 85%, and no adverse events were reported. These findings demonstrate that a structured telehealth lifestyle program is a safe, effective, and sustainable way to manage pediatric obesity, matching traditional in-person care in improving weight, behaviors, and quality of life [347,348,349]. In addition, Hagman et al. describe a 3-year pragmatic clinical trial evaluating the long-term effects of adding a digital treatment tool (Evira) to standard pediatric obesity care [350]. The Evira system integrated a digital home scale, mobile app, and web-based clinic interface to provide ongoing feedback and communication, along with regular in-person visits. The digital and physical (“digi-physical”) approach resulted in significantly greater weight loss and higher obesity remission rates over three years compared to standard care alone (BMI Z-score change: −0.29 vs. −0.12 (Evira vs. standard care), *p* = 0.02, obesity remission: 31.8% vs. 18.7%, *p* = 0.0046).

A number of longitudinal pediatric studies demonstrate that early intervention, particularly through lifestyle modifications that combine diet and exercise, can reverse early vascular and metabolic changes. The most convincing evidence comes from studies measuring carotid IMT and endothelial function, key early events in atherogenesis that precede plaque formation. In a randomized trial of 82 overweight children (body mass index, 25 ± 3; ages 9–12 years) at 6 weeks, both interventions were associated with decreased waist-to-hip ratio (*p* < 0.02) and cholesterol levels (*p* < 0.05), as well as improved arterial endothelial function [351]. Diet combined with exercise showed significantly greater improvement (*p* = 0.01). At 1-year follow-up, children who continued to exercise (*n* = 22) demonstrated substantially less carotid wall thickening (*p* < 0.001) and sustained improvements in vascular function compared to those who discontinued exercise (*n* = 19). Donghui et al. followed 57 male adolescents with BMI above 23.9 over a 6-week period of intervention with diet and exercise and found significantly decreased weight, BMI, LDL, triglycerides, insulin, and diastolic blood pressure [352]. Beneficial changes in vascular endothelial function were documented by measuring the Reactive Hyperemia Index. In a pediatric study from Italy, 55 children aged 6 through 16 years with obesity were evaluated before and after a 12-month behavioral program based on the Mediterranean diet plus an exercise regimen. After 12 months, those who lost weight demonstrated an increase in the mitral peak early diastolic velocity and a reduction in left ventricular area and volume. Intervention also reduced carotid IMT and decreased systemic blood pressure in the subjects [353].

Unfortunately, many adolescents with severe obesity do not achieve significant weight loss through lifestyle modifications alone. Inadequate results can occur, and those who do not experience sufficient weight reduction with lifestyle changes may benefit from pharmacotherapy or surgical intervention [354,355].

### 7.3. Role of Vitamin D in Pre-Diabetes Progression

The role of vitamin D in preventing the progression of pre-diabetes or type 2 diabetes is currently being actively studied [356,357,358,359]. Vitamin D is involved in the regulation of insulin secretion and affects tissue sensitivity to insulin. Vitamin D also has anti-inflammatory and cardio-protective properties [360,361]. It promotes insulin release by directly binding to vitamin D receptors located in pancreatic β-cells or indirectly increasing plasma calcium levels to activate calcium-dependent insulin release [362]. Vitamin D deficiency is prevalent among children, especially those who are obese. This might be attributed to a sedentary lifestyle that limits sun exposure and poor dietary habits. Additionally, the fat-soluble nature of vitamin D and the increased demand for this vitamin during puberty may also contribute to this deficiency [363].

A previous meta-analysis showed that vitamin D supplementation improved HOMA-IR (Homeostatic Model Assessment of Insulin Resistance). A high daily dose (≥4000 IU/d) of vitamin D may reduce CRP and increase HDL levels [364]. Another recently published meta-analysis suggests that vitamin D supplementation may have beneficial effects on glycemic, lipid, and inflammatory markers in patients with diabetes and prediabetes. Specifically, it significantly reduces HbA1c%, HOMA-IR, LDL cholesterol, total cholesterol, triglycerides, fasting insulin, and fasting glucose, while lowering C-reactive protein levels. Additionally, vitamin D supplementation increases the rate of normoglycemia among individuals with prediabetes [365]. Additional research is essential to enhance the evidence supporting the role of vitamin D in managing diabetes. explained.

### 7.4. Pharmacotherapy for Obesity and Pre-Diabetes

#### 7.4.1. Metformin

It is essential to review the use of the oral anti-hyperglycemic agent metformin when discussing the pharmacotherapy of exogenous obesity, type 2 diabetes, pre-diabetes, and insulin resistance [366]. Metformin is FDA-approved in children 10 years and older with type 2 diabetes [367]. Metformin decreases glucose production by the liver, improves insulin sensitivity, and may decrease appetite. A study from Adeyemo et al. found an average improvement in BMI of only 1.16 kg/m^2^ over 6–12 months [368]. In close agreement, a meta-analysis of clinical trials indicated that metformin reduced BMI by 1.07 kg/m^2^ [369]. Gastrointestinal disturbances are the most commonly reported adverse effects among patients. It is important to monitor these patients for vitamin B12 deficiency and lactic acidosis. Additionally, the 2016 guidelines from the American Association of Clinical Endocrinologists and the American College of Endocrinology recommend using metformin for patients with obesity who show signs of pre-diabetes or insulin resistance that do not improve with lifestyle modifications [370].

Bee et al. report that a stepped care diabetes prevention program, which included lifestyle changes, metformin, and financial incentives, reduced the 3-year diabetes risk by about 11% in a multiethnic Asian prediabetes cohort. In the intervention arm, 26.4% of participants received metformin [371].

#### 7.4.2. GLP-1 Agonists

A discussion of obesity management at present must consider glucagon-like peptide-1 (GLP-1) agonists. GLP-1 is a hormone secreted from the L-cells of the ileum and colon in response to food intake. It also improves insulin secretion from the pancreas and delays gastric emptying, therefore decreasing appetite [372,373]. GLP-1 agonists bind to the GLP-1 receptor, stimulating adenyl cyclase, which results in insulin secretion stimulated by glucose, inhibition of glucagon secretion, and slowing of gastric emptying, all of which permit better glycemic control. The most common adverse effects were of gastrointestinal origin [374]. GLP-1 agonists were FDA-approved and found to be effective in adolescents with exogenous obesity aged 12 years and older [375,376]. Liraglutide and semaglutide, both GLP-1 receptor agonists, have shown effectiveness in weight reduction and weight loss maintenance in children [377,378]. Liraglutide is a short-acting GLP-1 agonist approved for the treatment of type 2 diabetes in children aged 10 years and older and approved for weight loss for obese children aged 12 years and above [379,380,381]. A recent randomized placebo-controlled study over 56 weeks found liraglutide to be effective for weight loss in children from age 6 to below age 12 years, with an average decrease in BMI of 5.8% [382]. A meta-analysis by Kotecha et al. involving 18 randomized trials with youths aged 6 to 17 years who were treated with GLP-1 agonists for diabetes or obesity demonstrated effectiveness in reducing BMI across both the overall population and the obesity subgroup [383]. Semaglutide, a longer-acting GLP-1 agonist, was approved for the treatment of pediatric obesity in children aged 12 years and older and for the management of type 2 diabetes in patients aged 18 years and older [384]. There is a reported 4% risk of cholecystitis in patients treated with semaglutide in the STEP TEENS study [375]. These medications are not advised for individuals with a personal or family history of medullary thyroid carcinoma or multiple endocrine neoplasia type 2, due to concerns about C-cell tumors observed in rodents [385,386]. On a positive note, GLP1-receptor activation is implicated in anti-inflammatory pathways. Cardiovascular, kidney, and chondrocyte protective effects were also demonstrated [387,388]. They also improve blood pressure control [389,390]. Several GLP-1 agonists, such as liraglutide, once weekly semaglutide, and dilaglutide, have been associated with a reduction in atherosclerotic cardiovascular events in adults [391,392].

Ford et al., in their pediatric weight management multicenter study, demonstrated the importance of weight loss by showing that an improvement in BMI by 0.25 standard deviations was associated with improvements in the total cholesterol/HDL ratio, and mean systolic and diastolic blood pressure [393]. In this study, a decrease in BMI by 0.5 standard deviations led to a significant reduction in triglycerides (~30%), LDL-cholesterol (~15%), and CRP (~45%), again stressing the importance of timely treatment of obesity in preventing cardiovascular complications.

#### 7.4.3. Other Incretin-Based Therapies

Tirzepatide, a combination GLP-1 and glucose-dependent insulinotropic polypeptide 1 (GIP1) agonist, is approved for the treatment of obesity in patients aged 18 years and older [394]. The dual treatment led to a mean body weight reduction at 176 weeks of 12.3% in adult patients and delayed progression to type 2 diabetes [395,396]. Tirzepatide continues to create excitement for the development of anti-obesity medications and has shown efficacy in children with obesity and diabetes [397].

Retatrutide, a triple receptor GLP-1, GIP, and glucagon receptor agonist, is used in adults with obesity. At 48 weeks, a weight reduction of 5% or more, 10% or more, and 15% or more had occurred in 100%, 93%, and 83%, respectively, of those who received 12 mg (the highest dose); compared to 27%, 9%, and 2% weight reduction in those who received placebo [398]. The most common side effects were gastrointestinal, which were dose-dependent. Retatrutide is currently not approved for children. However, a clinical trial (NCT06075667) is underway studying once weekly retatrutide in adolescents aged 12 to 17 years who are obese or overweight with weight-related comorbidities [399].

Consuming protein-rich foods and instituting a regimen of weight-bearing exercise is essential for patients who are on these medications because they can deplete muscle mass and this may have severe consequences in growing and developing youth [400,401].

A recent publication in the New England Journal of Medicine detailed a Phase 2 trial of Maridebart cafraglutide, also known as MariTide or AMG133 (developed by Amgen) [402]. This treatment consists of two identical GLP-1 peptide analogs that are linked to a single monoclonal antibody that acts as an antagonist for the GIP receptor [403]. It has a half-life of about 21 days, roughly three times longer than the longest-acting FDA-approved once-weekly obesity medications, supporting monthly or less frequent dosing. The phase 2 study involved adult patients with obesity, both with and without type 2 diabetes. Participants experienced an average weight loss of up to 16.2%. Those with type 2 diabetes achieved an average weight reduction of 12.3% and saw a decrease in glycated hemoglobin levels of up to 1.6%. The most commonly reported side effects were gastrointestinal in nature and depended on the dosage taken. The weight plateau was not observed at 52 weeks, as weight continued decreasing. Longer-term studies are needed to fully evaluate the efficacy of this agent.

Use of incretin-based therapies is not without consequences and can lead to serious side effects, with pancreatitis a major gastrointestinal concern [404,405]. Use of GLP-1 agonists and DPP-4 inhibitors in patients with type 2 diabetes may lead to pancreatitis [406]. Other reported side effects include nausea and vomiting, resulting from the activation of specific GLP-1 receptors in the hindbrain [407].

#### 7.4.4. Other Diabetes Therapies

Regarding specifically type 2 diabetes management, in addition to GLP-1 agonists, sodium-glucose cotransporter 2 (SGLT2) inhibitors such as dapaglifozin, act by enhancing renal glucose excretion and are safe and efficacious in children and adolescents [408,409]. Although data is sparse, thus far SGLT2 inhibitors have not been found to bring about weight loss in youth with type 2 diabetes [410].

Dipeptidyl peptidase-4 (DPP4) inhibitors (vildagliptin, linagliptin, saxagliptin, and sitagliptin) work by inhibiting the inactivation of GLP-1 and GIP by the DPP-4 enzyme, allowing GLP-1 and GIP to potentiate the secretion of insulin in β cells and suppress glucagon release by α cells [411]. Clinical studies investigating the effect of the oral anti-hyperglycemic DPP-4 inhibitor vildagliptin on patients with impaired glucose tolerance showed improvement in β-cell function and postprandial glycemia [412,413]. When combined with metformin, vildagliptin enhanced weight loss beyond that of metformin alone in adults with type 2 diabetes [414]. Use of DPP-4 inhibitors is not recommended in children.

#### 7.4.5. Phentermine

The combination of phentermine/topiramate extended release was approved by the FDA in 2022 for adolescents with obesity aged 12 years and older. This combination provides both immediate release phentermine for appetite reduction early in the day, and extended release topiramate for appetite reduction later in the day and into the evening [415]. A 56-week randomized clinical trial with 227 participants aged 12–17 years reported a 10.44% change in BMI (95% CL, −13.89% to −6.99%) for the highest dose of phentermine/topiramate [416]. The safety profile was acceptable, but worsening of depression and suicidal ideation were a concern in the study group. The most commonly reported adverse events included paresthesia, dry mouth, constipation, dysgeusia, and insomnia, which were observed at higher percentages in the high-dose group compared to both the low-dose group and the placebo. Phentermine/topiramate extended release.is considered a cost-effective treatment [417,418].

#### 7.4.6. Orlistat

It is also worth mentioning that one of the older medications approved for pediatric obesity is orlistat, which inhibits fat absorption by blocking pancreatic and gastric lipases. The recommended dose for children 12 years and older is 120 mg given 3 times per day with meals [419]. It has only modest effects and is often poorly tolerated due to its side effects of nausea, loose bowel movements, oily stools, abdominal pain, bloating, and fecal incontinence. There is also a concern about loss of fat-soluble vitamins (A, D, E, K) [420,421].

#### 7.4.7. Medications for Genetic Forms of Obesity

Genetic forms of obesity fall into a separate category and are addressed here, with an emphasis on ways in which genetic predispositions affect body weight regulation, fat distribution, insulin sensitivity, and glucose metabolism—key factors in the development of pre-diabetes and type 2 diabetes. Additionally, because disruptive food-seeking behaviors in some individuals lead to poor adherence to lifestyle modifications, conservative approaches like lifestyle changes are often less effective, making weight loss more challenging for this group and increasing their risk of pre-diabetes and type 2 diabetes [422].

The leptin-melanocortin pathway is responsible for appetite control [423]. Leptin, synthesized by the leptin gene (LEP) in adipocytes, activates its receptor (LEPR), inducing prohormone convertase 1/3 (PCSK1) activity in anorexigenic neurons, which converts proopiomelanocortin (POMC) to alpha-melanocyte-stimulating hormone (α-MSH) [424]. α-MSH binds the melanocortin receptor type 4 (MC4R), which induces a satiety signal upon activation [425]. Recently, a cyclic peptide and highly selective MC4R agonist, setmelanotide, was synthesized [426]. Daily subcutaneous injection of setmelanotide for one year resulted in significant appetite control, consequently leading to weight loss in a trial assessing POMC and LEPR deficient patients stemming from POMC, PCSK1, or LEPR homozygous mutations [427]. The FDA approved setmelanotide in November 2020 for the treatment of children older than 6 years with genetic mutations that are likely pathogenic or of uncertain significance, causing LEPR, POMC, or PCSK1 deficiency [428]. In June 2022, setmelanotide was approved for use in individuals with Bardet-Biedl Syndrome, a rare autosomal recessive disorder that is genetically heterogeneous and associated with hyperphagia and obesity [429]. Reaction at the injection sites, skin hyperpigmentation, rash, alopecia, gastrointestinal disturbance, and flu-like symptoms were reported adverse reactions [430].

Prader–Willi Syndrome (PWS), another rare disorder, is paternally inherited via chromosome 15q11.2–q13 and characterized by compulsive food consumption with failure of satiety and morbid obesity [431]. PWS patients exhibit low muscle tone and failure to thrive in infancy, then obesity and hyperphagia occur later in childhood. The timely management of obesity with the goal of prevention of pre-diabetes and type 2 diabetes is critical in PWS, because prevalence of type 2 diabetes in these patients (7–24%) is much higher than in the general population (5–7%) [432]. Patients with PWS have a decreased number of oxytocin-producing neurons, which affects social skills, food intake, energy expenditure, and body weight regulation [433,434]. Intranasal oxytocin showed promising results in the youngest age group [435,436]. However, more studies are needed to further understand the role of oxytocin in treating patients with PWS [437].

Hyperphagia in PWS likely occurs due to impaired leptin activation of potassium ATP channels, which results in increased secretion of neuropeptide Y by the neurons in the arcuate nucleus. Therefore, it was hypothesized that stimulating these channels in the neurons with diazoxide choline might magnify the effect of leptin by decreasing secretion of neuropeptide Y [438]. A clinical trial of diazoxide choline treatment of 11 patients with PWS at the University of California, Irvine, found improvements in hyperphagia, resting energy expenditure, body fat reduction, and an increase in the lean body mass to body fat ratio. Unfortunately, there was no significant change in weight from baseline to the end of the study [439]. A recent publication by Miller et al. had similar results [440]. In Miller et al. study, improvements were observed in aggression, anxiety, and compulsivity. There were reductions in leptin, insulin, and insulin resistance, along with a significant increase in adiponectin. Lean body mass was also increased. All results were statistically significant. In March 2025, the U.S. FDA approved diazoxide choline in extended release form for treating hyperphagia in patients aged 4 years and older with PWS [441].

### 7.5. Metabolic and Bariatric Surgery

The Endocrine Society, the American Academy of Pediatrics and the American Society of Metabolic and Bariatric Surgery [17,338,442] have all published guidelines that include metabolic and bariatric surgeries as an option for pediatric patients with severe obesity. These criteria are met if the patient has attained Tanner 4 or 5 pubertal development, reached final or near-final adult height, and has a BMI of 40 kg/m^2^ or a BMI of 35 kg/m^2^ with significant extreme comorbidities. Extreme obesity and comorbidities must also persist despite healthy lifestyle modifications, with or without pharmacotherapy, to be eligible for surgery. The patient must be psychologically stable without an underlying, untreated mental disorder and show adherence to the principles of healthy dietary and lifestyle habits after surgery [17,443].

Laparoscopic sleeve gastrectomy and Roux-en-Y gastric bypass (RYGB) are the most common procedures, with the latter being the most frequent [444]. RYGB led to the most significant weight reduction in various studies at 1 and 5-years post-operation [445]. In a review from Vuong et al., females aged 17–19 years represented the majority of patients [446]. Additionally, Vuong et al. reported no inpatient mortality, and the complication rate was 1% or less. Data suggests postsurgical deficiencies in micronutrients like iron deficiency (45–71%), vitamin B12 deficiency (20%), vitamin D deficiency (41%), and effects on long term bone health were potential concerns in these patients [447]. Shacker et al. analyzed outcomes of RYGB and sleeve gastrectomy using a multicenter, national database of a large cohort of patients with 2% categorized as adolescents (10–19 years of age). Compared to young adults (age 20 to 29 years), the adolescent group experienced fewer postoperative complications, including surgical site infections, gastrointestinal bleeding, and blood transfusions (*p* < 0.001), as well as fewer readmissions. Sleeve gastrectomy was the most common procedure in the youngest group [448].

## 8. Conclusions and Future Directions

Prediabetes in children signals underlying metabolic vulnerabilities shaped by a complex interplay of factors such as obesity, insulin resistance, polycystic ovarian syndrome (PCOS), genetic predispositions, intrauterine exposures, and sociodemographic influences. Comparability across studies of prediabetes and obesity in children is significantly limited by inconsistent diagnostic criteria and classification systems, creating challenges for interpreting prevalence data and treatment outcomes. Effective disease prevention demands several key prerequisites, including established diagnostic criteria, understanding of risk factors and the disease’s natural progression, as well as affordable and acceptable screening methods to identify individuals at high risk. Tailoring the diagnosis of prediabetes by considering both pathophysiology and clinical characteristics can help identify the risk for type 2 diabetes and CVD, thereby enhancing the effectiveness of intervention strategies. It is crucial to address the risk factors associated with prediabetes in children, as this condition can lead to numerous complications. Beyond type 2 diabetes, these complications include hypertension, CVD, fatty liver disease, and sleep apnea, all of which are becoming more common among children.

Achieving therapeutic success requires a proactive and collaborative effort that goes beyond the clinic, combining early and sustainable lifestyle changes, targeted screening, and family-centered care. Intensive lifestyle modification therapy, pharmacotherapy, and bariatric/metabolic surgery are used in children and adolescents and have shown efficacy. This approach engages a multidisciplinary team comprising nutritionists, physicians, psychologists, other specialists, and community stakeholders to address the diverse needs of at-risk children and create environments where healthier lives can thrive. These interventions have demonstrated short-term benefits in reducing the incidence of type 2 diabetes. In the long term, however, more dedicated research on pediatric prediabetes is needed to improve future health outcomes.

## Figures and Tables

**Table 1:** Risk factors for developing prediabetes in the pediatric age group.

**Risk Factor**	**Description**	**References**
Overweight or obesity	BMI ≥ 85th percentile (overweight) or ≥95th percentile (obesity)	[17,18,26]
Family history of type 2 diabetes	First- or second-degree relative with type 2 diabetes	[17,18,22,27]
Maternal history of diabetes or gestational diabetes	Diabetes or gestational diabetes mellitus during the child’s gestation	[25]
High risk race/ethnicity/ancestry	African American, Hispanic/Latino, Native American, Asian American, Pacific Islander	[22,28,29]
Signs of insulin resistance or related conditions	Acanthosis nigricans, hypertension, dyslipidemia, polycystic ovary syndrome, abnormal birth weight, chronic stress, sleep disorders	[18,30,31]
Sedentary lifestyle/physical inactivity	Low physical activity contributing to obesity and insulin resistance	[19]
Pubertal status	Physiologic insulin resistance of puberty increases risk	[18]
Maternal obesity	Maternal obesity during pregnancy independently confers elevated risk	[18]
Genetic susceptibility	Specific genetic variants, family history, and ethnic predisposition	[17,19]

**Table 2:** Major risk factors for development of cardiovascular disease.

Category	Risk Factors	References
Non-modifiable	Age, sex (male), family history of premature atherosclerotic cardiovascular disease, race, and ethnicity (e.g., South Asian ancestry)	[275]
Modifiable—Cardiometabolic factors	Hypertension, diabetes, dyslipidemia (high LDL, non-HDL cholesterol), high triglycerides, serum ratio of ApoB/ApoA1	[275]
Modifiable-Lifestyle factors	Physical inactivity, diet, obesity, smoking, sleep pattern, alcohol consumption, depression/psychosocial stress, low socioeconomic status	[276,282]

HDL: high-density lipoprotein; LDL: low-density lipoprotein.

## Data Availability

No new data were created or analyzed in this study. Data sharing is not applicable to this article.
